# Semaphorin 3F and Neuropilin-2 Control the Migration of Human T-Cell Precursors

**DOI:** 10.1371/journal.pone.0103405

**Published:** 2014-07-28

**Authors:** Daniella Arêas Mendes-da-Cruz, Anne Colette Brignier, Vahid Asnafi, Frederic Baleydier, Carolina Valença Messias, Yves Lepelletier, Nawel Bedjaoui, Amedée Renand, Salete Smaniotto, Danielle Canioni, Pierre Milpied, Karl Balabanian, Philippe Bousso, Stéphane Leprêtre, Yves Bertrand, Hervé Dombret, Norbert Ifrah, Mireille Dardenne, Elizabeth Macintyre, Wilson Savino, Olivier Hermine

**Affiliations:** 1 CNRS UMR8147, Paris Descartes University, Paris, France; 2 Oswaldo Cruz Institute, Oswaldo Cruz Foundation, Rio de Janeiro, Brazil; 3 Department of Clinical Hematology, Necker Children's Hospital, Assistance Publique-Hôpitaux de Paris (AP-HP), Paris, France; 4 Laboratory of Oncohematology, AP-HP, Necker Children's Hospital, INSERM U1151, Paris, France; 5 INSERM U1163, CNRS ERL 8254, Laboratory of cellular and molecular basis of hematological disorders and their therapeutic implications, Imagine Institute, Paris, France; 6 Department of Morphology, Federal University of Alagoas, Maceió, Brazil; 7 Department of Pathology, Necker Hospital, Paris, France; 8 INSERM U819 – Pasteur Institute, Paris, France; 9 INSERM U668 – Pasteur Institute, Paris, France; 10 Department of Hematology, Centre Henri Becquerel, Rouen, France; 11 Service of Pediatric Hematology, Hôpital Debrousse, Lyon, France; 12 University Paris 7, Hôpital Saint-Louis, AP-HP, and Institut Universitaire d'Hématologie EA3518, Paris, France; 13 Pôle de Recherche et d'Enseignement Supérieur l'Université Nantes Angers Le Mans, Centre Hospitalier Universitaire Angers, Service des Maladies du Sang and INSERM U892, Angers, France; Universidade de Sao Paulo, Brazil

## Abstract

Neuropilins and semaphorins are known as modulators of axon guidance, angiogenesis, and organogenesis in the developing nervous system, but have been recently evidenced as also playing a role in the immune system. Here we describe the expression and role of semaphorin 3F (SEMA3F) and its receptor neuropilin-2 (NRP2) in human T cell precursors. NRP2 and SEMA3F are expressed in the human thymus, in both lymphoid and non-lymphoid compartments. SEMA3F have a repulsive effect on thymocyte migration and inhibited CXCL12- and sphingosine-1-phosphate (S1P)-induced thymocyte migration by inhibiting cytoskeleton reorganization prior to stimuli. Moreover, NRP2 and SEMA3F are expressed in human T-cell acute lymphoblastic leukemia/lymphoma primary cells. In these tumor cells, SEMA3F also blocks their migration induced by CXCL12 and S1P. Our data show that SEMA3F and NRP2 are further regulators of human thymocyte migration in physiological and pathological conditions.

## Introduction

Thymocyte migration is critical for normal T cell development and maturation. From the entrance of precursors into the thymus, to the migration within the organ and finally mature thymocyte egress, several molecules and receptors are implicated, including extracellular matrix (ECM) molecules, chemokines, sphingosine-1-phosfate (S1P) and their respective receptors. ECM proteins such as fibronectin and laminin are present in the thymus in different concentrations depending on the region. They are recognized by integrins constitutively expressed on thymocytes and microenvironmental cells. The ECM-integrin interactions induce cell adhesion and migration, and also mediate cell-cell interactions [1]. Chemokines are well described in the thymus, playing a role in all migratory steps described above. One classical chemokine described as being chemoattractant or chemorepellent for thymocytes, depending on the dose applied, is CXCL12, which binds its cognate receptor CXCR4 [2]. Despite normal thymus development and thymocyte differentiation in CXCR4^−/−^ mice, the emigration of mature CD4 thymocytes is severely impaired, and these cells are retained in the thymus [3]. In the human thymus, CXCR4 is also preferentially expressed in immature thymocytes and promote attraction of these cells [4], [5]. In addition, besides thymocyte attraction, CXCR4 seems to play a role in the retention of immature CD4^+^CD8^+^ double-positive (DP) cells in the cortex [6]. In a second vein, some studies also demonstrate the essential role of sphingosine-1 phosphate type 1 receptor (S1P_1_) and its ligands in thymocyte egress. S1P_1_-deficient precursors can differentiate normally within the thymus but are unable to exit the organ [7]. Mouse thymocytes upregulate S1P_1_ expression during differentiation, and therefore mature single-positive (SP) cells expressing higher levels of the receptor are able to respond to S1P gradients [8]. *In vivo*, small concentrations of the natural S1P_1_ ligand, (S1P) promote lymphocyte migration, whereas therapeutic concentrations of FTY720 (a synthetic S1P agonist) inhibit lymphocyte egress from the thymus and peripheral lymphoid organs, inducing a cell sequestration in those organs and a profound lymphopenia [9], [10], [11].

To add to the complexity of this regulatory network, some molecules initially described in the nervous system are also present in the thymus playing a role in T cell development and function. Neuropilins (NRPs) and semaphorins (SEMAs), initially known as modulators of axon guidance, angiogenesis, and organogenesis [12], [13] are also expressed in the immune system, and we have previously demonstrated that NRPs and SEMAs also play a role in thymocyte migration. Thymocytes and thymic epithelial cells express NRP1 and its ligand SEMA3A, which induces a reduction of thymocyte adhesion to thymic epithelial cells (TEC) and induced thymocyte chemorepulsion [14], [15]. During development, NRP2 is also expressed by cells from both central and peripheral immune system in mice including thymocytes and the thymic microenvironmental cells, as well as peripheral lymphocytes and macrophages [16], [17]. Although the expression of NRP2 and its ligand SEMA3F in the mouse thymus have been studied during development, their expression and functional role in the human thymus remained unknown. Herein, we show that NRP2 is strongly expressed in human thymocytes, which led us to study the functional role of this receptor and the SEMA3F ligand in thymocyte migration.

## Materials and Methods

### Human thymuses

Human thymuses were obtained as a by-product of cardiac surgery performed on children aged from one week to 11 years of age at Necker Hospital. Participants and next of kin, caretakers, or guardians on the behalf of the minors/children participants provided their written informed consent to participate in this study, which was approved by the Necker Hospital Ethical Committees for human research and were performed according to the European Union guidelines and the declaration of Helsinki.

### Antibodies and chemicals

The following antibodies were used in appropriate dilutions. APC/anti-CD4 and PerCP/anti-CD8 were purchased from Becton-Dickinson (South San Francisco, USA). Purified anti-neuropilin-2 and FITC/donkey anti-goat were obtained from Santa Cruz Biotechnology (Santa Cruz, USA), whereas purified anti-S1P_1_ and corresponding neutralizing peptide were from ABR Affinity BioReagents (Golden, USA). Rabbit anti-cytokeratin immunoserum was a Dako product (Carpinteria, USA) and rhodamine/goat anti-rabbit Ig was from Sigma-Aldrich (St Louis, USA). Purified anti-SEMA3F was a Chemicon International product (Temecula, USA). Secondary PE/donkey anti-goat and FITC/donkey anti-rabbit antibodies were from Jackson ImmunoResearch Laboratories Inc. (West Grove, USA).

Recombinant human CXCL12 and mouse SEMA3F were from R&D Systems, whereas S1P was a Sigma-Aldrich product.

### Cytofluorometry

Cells were washed and maintained in PBS for cell counting and subsequent staining. For intracellular staining, a PBS/BSA/saponin solution was used to dilute both primary and secondary antibodies. Cells were then evaluated by flow cytometry in a FACSCalibur or a FACSCanto II device (Becton Dickinson, San Jose, USA); analyses were done using a CellQuest or FACSDiva software (Becton-Dickinson).

### Real-time quantitative polymerase chain reaction (RQ-PCR)

Following mRNA isolation and cDNA synthesis, RQ-PCR was done with TaqMan Universal PCR Master Mix (Applied Biosystems, Foster City, USA) in a 7900HT Fast Real Time PCR System and analyzed in SDS 2.3 software (Applied Biosystems). Primers and probes were designed using the Primer Express software (Applied Biosystems). NRP2 sense: TGCCCAGCTACGACATGGA; NRP2 anti-sense: CAATCTCTCCGGAACGTCCTT; NRP2 probe CCAGATTGTGTTCGAGGGAGTGATAGGG; SEMA3F sense: TCGCCCCAAGCCACTGT; SEMA3F anti-sense: AGCGGTCCTCTGCACGAAT; SEMA3F probe: CAGCGAGATCCTGGTGACCGGC; S1P_1_ sense: GGCTCTCCGAACGCAACTT; S1P_1_ anti-sense: CAGGCTTTTTGTGTAGCTTTTCC; S1P_1_ probe: TTTCCGAGGCCCTCTCCAGCCA; ABL sense: TGGAGATAACACTCTAAGCATAACTAAAGGT; ABL anti-sense: GATGTAGTTGCTTGGGACCCA; ABL probe: CCATTTTTGGTTTGGGCTTCACACCATT. mRNA quality was assessed and normalized by quantification of Abelson (ABL) gene, using guidelines from the Europe Against Cancer program. Samples with an ABL cycle threshold (Ct) higher than 29 were excluded from analysis. Each experiment included two non-template controls for contamination and each sample was performed in duplicate.

### Immunofluorescence

Human thymuses were frozen and sectioned (4 µm), and slides were incubated with BSA 1% blocking solution. Samples were subjected to primary antibodies overnight at 4°C or 1 h at room temperature followed by corresponding secondary antibodies for 30 min at room temperature. Stained samples were analyzed by confocal microscopy using a LSM 5 Pascal device (Germany). Negative controls, in which primary antibodies were replaced by unrelated immunoglobulins, or in which the secondary antibody was used alone, did not generate any significant fluorescent signal.

### Transmigration assays

Cell migratory activity was assessed in a transwell system. Inserts bearing 5 µm pore size in transwell plates (Corning Costar, Cambridge, USA) were treated with PBS/BSA 1%. Cells (2.0 or 2.5×10^6^) in 100 µl of migration medium alone (RPMI/BSA 1%) or medium containing SEMA3F (R&D Systems - Minneapolis, MN, USA) as a chemorepulsive stimulus at 100 ng/ml were added in the upper chambers. After 4 h of incubation at 37°C in 5% CO_2_ humidified atmosphere, migration was defined by counting the cells that migrated to the lower chambers containing migration medium alone or the medium containing the chemoattractant molecules CXCL12 (200 ng/ml) or S1P (10 nM). Cells were then labeled with appropriate antibodies and analyzed by flow cytometry. In some experiments, cells were first incubated with anti-NRP2 blocking mAb (R&D Systems) before migration.

### Actin polymerization Assay

Cells were incubated in RPMI 1640 medium/20 mM HEPES. CXCL12 or SEMA3F were then added to the cell suspension. At each indicated time point (15 s to 2 min), an aliquot was taken from the cell suspension and mixed with the labeling buffer, consisting in 10^−7^M FITC-phalloidin (Sigma-Aldrich), 0.125 mg/ml L-alpha-phosphatidylcholine palmitoyl (Sigma-Aldrich), and 4.5% PFA in PBS. Staining was analyzed by flow cytometry. Mean fluorescence intensity (MFI) values obtained before addition of ligand were arbitrarily set at 100%.

### Patients and samples

Diagnosis was made on peripheral blood or bone marrow T-cell acute lymphocyte leukemia (T-ALL) samples (n = 136), as previously described (Asnafi et al. 2004). Tumor diagnostic samples from 52 patients with T-cell lymphoblastic lymphoma (T-LBL) were available for molecular analysis. All patients provided informed consent in accordance with the Declaration of Helsinki, and the study was in accordance with local and multicenter research ethical committee approval.

### Statistical analyses

Results were analyzed by the parametric Student's *t* test, one-way ANOVA or the nonparametric Wilcoxon Mann-Whitney test. Differences were considered to be statistically significant when p<0.05 (*), p<0.01 (**) or p<0.001 (***).

## Results

### NRP2 and SEMA3F are expressed in the human thymus

**Figure 1:**
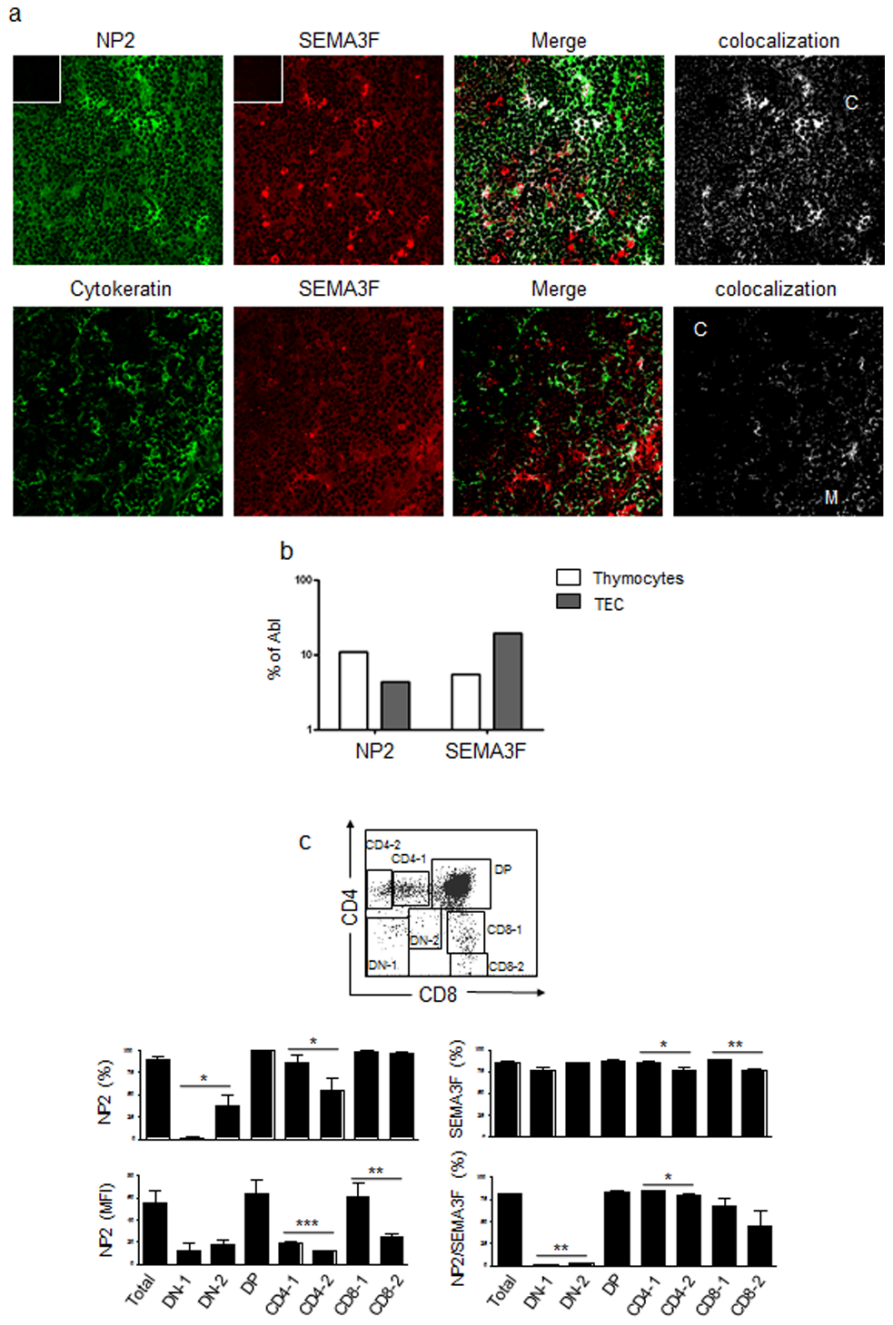
Expression of NRP2 and SEMA3F in the human thymus and thymocytes. **a)** Upper panels show the expression of NRP2 and SEMA3F in the human thymus *in situ*, ascertained by immunofluorescence and confocal microscopy analysis. Lower panels show the expression of SEMA3F and cytokeratin, revealing that SEMA3F is expressed in the epithelial as well as in the non-epithelial compartments of the thymus. Colocalization analysis was performed with ImageJ software. Inserts show negative controls for each secondary antibody. C: cortex; M: medulla. Magnification: 400×. **b)** NRP2 and SEMA3F mRNA expression analyzed by real time quantitative PCR, compared with the control Abelson (Abl) gene in fresh thymocytes and the THPN thymic epithelial cell line. **c)** Cytofluorometric dot plot depicts the regions used to separate the CD4/CD8-defined thymocyte subpopulations. Graphs represent the expression of NRP2 and SEMA3F in total thymocytes and each subpopulation. n = 3–6. In the case of NRP2, mean fluorescence intensity (MFI) analyses are shown to illustrate differences in the expression among the thymocyte subpopulations. Data are represented as means ± SEM. Selected thymocyte subsets were analyzed by the unpaired Student's *t* test and differences were considered statistically significant when p<0.05 (*), p<0.01 (**) or p<0.001 (***). DN-1: CD4^−^CD8^−^ cells; DN-2: CD4^low^CD8^low^; DP: CD4^+^CD8^+^; CD4-1: CD4^low^CD8^−^; CD4-2: CD4^high^CD8^−^; CD8-1: CD4^−^CD8^low^; CD8-2: CD4^−^CD8^high^.

We first observed that NRP2 and SEMA3F were constitutively expressed in developing human T cells in the thymus. The expression of both NRP2 and SEMA3F was widely observed in the epithelial cells (defined by cytokeratin staining) as well as in non-epithelial components in thymic sections (Fig. 1a), as well as in primary TEC cultures and a TEC cell line (data not shown). mRNA expression of corresponding transcripts was also quantified on thymocytes and in a TEC line (Fig. 1b).

The expression of NRP2 on thymocytes varied according to the CD4/CD8-defined subpopulation. A very low percentage of CD4-CD8- double-negative (DN) thymocytes expressed NRP2, whereas almost all DP cells expressed this receptor (Fig. 1c). NP2 expression was lower in single positive (SP) CD4^−^CD8^+^ and CD4^+^CD8^−^ cells as they become CD4^high^CD8^−^ or CD4^−^CD8^high^ (Fig. 1c). SEMA3F was also expressed by all thymocyte subpopulations, but reduced percentages were observed in the CD4^high^CD8^−^ and CD4^−^CD8^high^ cells. Interestingly, the same tendency was observed in cells stained for both NRP2 and SEMA3F molecules (Fig. 1c). It is important to note that the expression of both molecules was not related to the children's sex or age (data not shown).

### SEMA3F and NRP2 play a role on thymocyte migration

**Figure 2:**
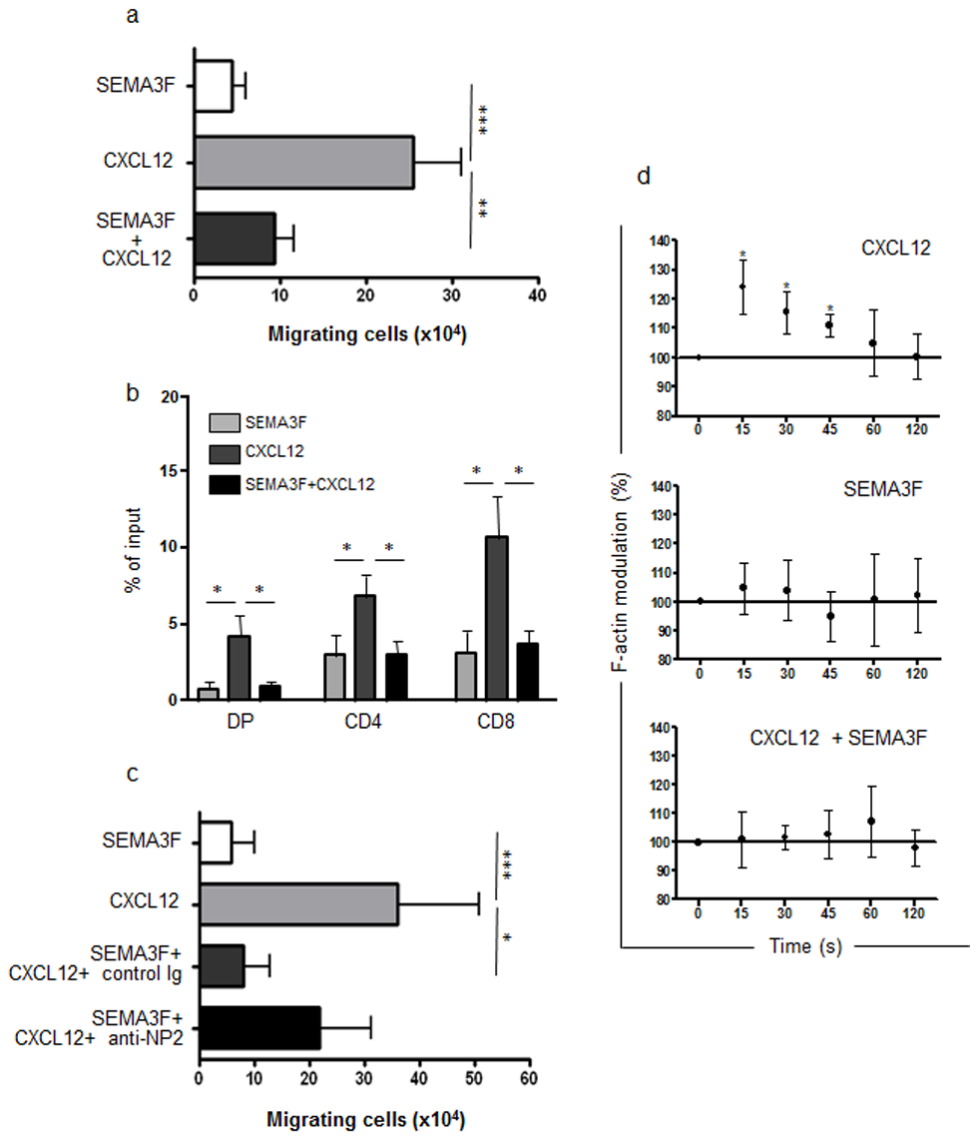
SEMA3F is repulsive and impairs the migratory response of human thymocytes towards CXCL12. **a)** Bars represent the numbers of migrating thymocytes in a transwell system. SEMA3F was added in the upper chambers to evaluate the repulsive response or blocking stimulus of thymocytes. CXCL12 was added in the bottom chambers and induced thymocyte migration. When both stimuli where combined, SEMA3F inhibited CXCL12-induced thymocyte migration (n = 10). **b)** Migration response of thymocyte subsets (defined by CD4/CD8 expression), showing that SEMA3F has effect in all thymocyte subpopulations. DP = double-positive, CD4 =  CD4 single positive, CD8 =  CD8 single-positive. **c)** Bars show the numbers of migrating thymocytes. Black bar represent thymocyte migration of cells pre-treated with anti-NRP2 blocking antibody which abrogated SEMA3F action, since the difference observed between CXCL12 and CXCL12+SEMA3F+anti-NRP2 is no longer significant (n = 3). Results were analyzed by the One-way ANOVA analysis of variance and Tukey's multiple comparison post-test. **d)** F-actin modulation of human thymocytes (n = 4–5) was analyzed by flow cytometry and shown herein as [(MFI after addition of ligand)/(MFI before addition of ligand)]×100. MFI values obtained before addition of ligand were arbitrarily set at 100% that corresponds to time zero. Data are represented as means ± SEM. Results were analyzed by the unpaired Student's *t* test, comparing each time point compared with time zero. Differences were considered statistically significant when p<0.05 (*), p<0.01 (**) or p<0.001 (***).

SEMA3F was first described as being chemorepulsive in the nervous system [18], and we observed a similar function in normal human thymocytes (Fig. 2a–c). When SEMA3F was added to the upper chambers of the transwell plates together with thymocytes, cells migrated to the lower chambers, in the opposite direction of the SEMA3F gradients (Fig. 2a). No migration was observed when this molecule was added to the lower chambers as a chemoattractant stimulus (data not shown).

In contrast, CXCL12, acting through its receptor, CXCR4, is known to reduce axonal responsiveness to several known repulsive molecules, including SEMA3A [19]. Since CXCL12 is an important thymocyte chemoattractant and thymocyte migration can be under control of a variety of simultaneous molecular interactions [20], we tested the effect of SEMA3F together with CXCL12. In this case, the presence of SEMA3F inhibited CXCL12-induced migration (Fig. 2a). Interestingly, this inhibition was observed not only in immature DP but also in the mature CD4 and CD8 SP thymocyte subpopulations (Fig. 2b).

Pre-treatment of thymocytes with the blocking anti-NRP2 mAb reverted the inhibition of CXCL12 chemoattraction caused by SEMA3F, showing that the response was specifically mediated by NRP2/SEMA3F interactions (Fig. 2c).

Because SEMA3F modulated thymocyte migration, we analyzed the regulation of the cytoskeleton following CXCL12 and SEMA3F stimulus. As shown in Figure 2d, CXCL12 promoted a peak of actin polymerization after 15 s of stimulus, while SEMA3F induced a slight modification. However, when cells exposed to CXCL12 were pre-treated with SEMA3F, the CXCL12-induced actin polymerization was prevented, showing that SEMA3F is able to inhibit the early steps of CXCL12 stimulus that leads to thymocyte migration.

### SEMA3F inhibits S1P-induced thymocyte migration

**Figure 3:**
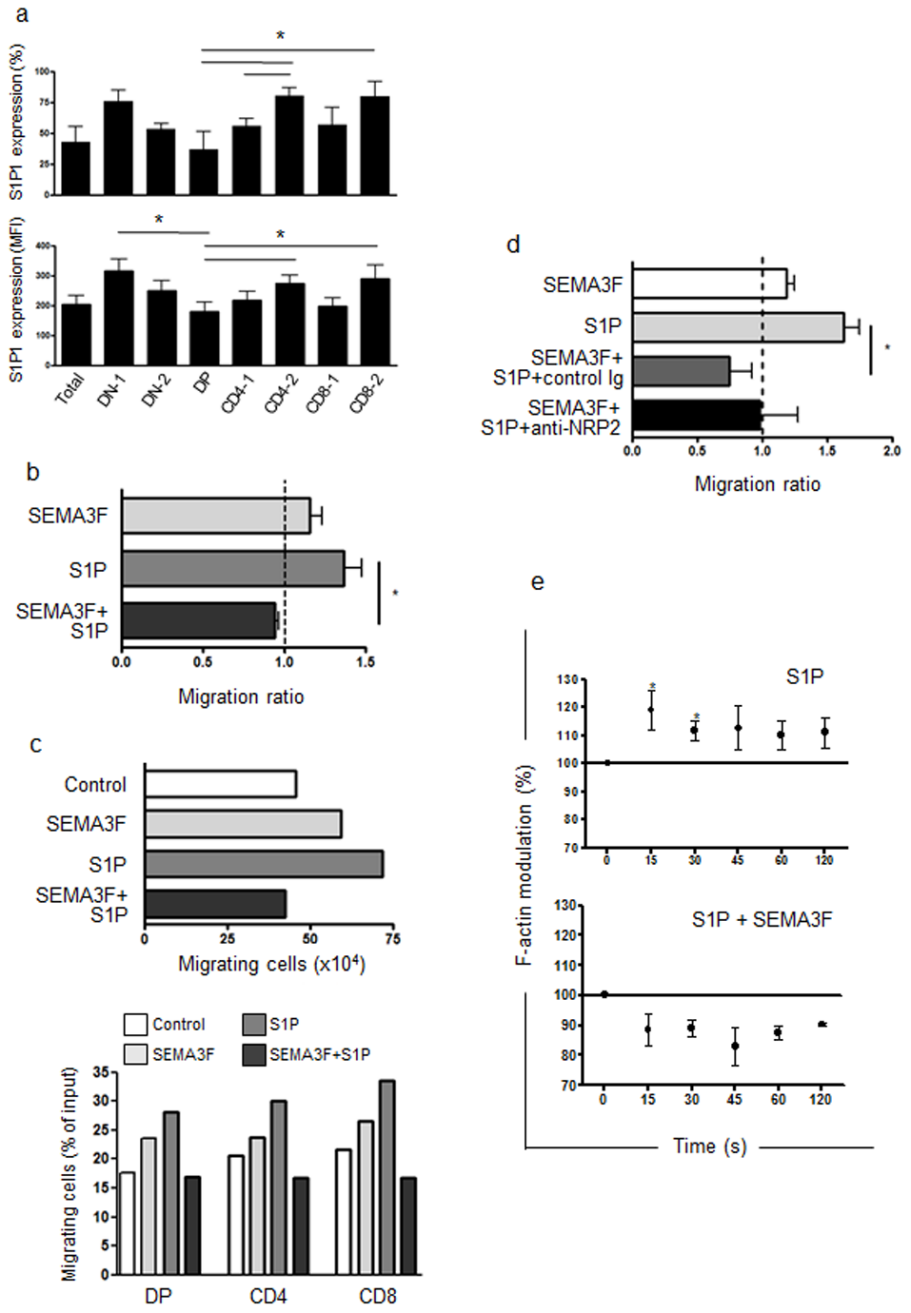
SEMA3F impairs the migratory response of human thymocytes towards S1P. **a)** S1P_1_ expression ascertained by flow cytometry in total human thymocytes and CD4/CD8-defined subsets as shown in Figure 1c. DN-1: CD4^−^CD8^−^ cells; DN-2: CD4^low^CD8^low^; DP: CD4^+^CD8^+^; CD4-1: CD4^low^CD8^−^; CD4-2: CD4^high^CD8^−^; CD8-1: CD4^−^CD8^low^; CD8-2: CD4^−^CD8^high^. n = 4. **b)** Bars represent migration of thymocytes in a transwell system. Results are represented by migration ratio, and controls without stimuli were normalized to 1.0. n = 4. Cells migrate towards S1P and when SEMA3F was combined with S1P, it inhibited S1P-induced thymocyte migration. **c)** Migration response of thymocytes from a representative experiment. Bottom panel show the migration of thymocyte subpopulations based on CD4/CD8 expression, showing that SEMA3F had effect and impaired S1P-induced migration in all thymocyte subpopulations. DP =  double-positive, CD4 =  CD4 single positive, CD8 =  CD8 single-positive. **d)** Bars show the numbers of migrating thymocytes. The black bar represent thymocyte migration of cells pre-treated with anti-NRP2 blocking antibody which partially abrogated SEMA3F action, as the difference observed between S1P and S1P+SEMA3F+anti-NRP2 is no longer significant (n = 3). **e)** F-actin modulation of human thymocytes (n = 4) was analyzed by flow cytometry and represented as [(MFI after addition of ligand)/(MFI before addition of ligand)]×100. MFI values obtained before addition of ligand were arbitrarily set at 100% that corresponded to time zero. Data are represented as means ± SEM.Results were analyzed by the unpaired Student's *t* test, comparing each time point with time zero. Differences were considered statistically significant when p<0.05 (*).

The role of S1P_1_ on thymocyte migration and egress has been well described in mice. Mouse thymocytes upregulate S1P_1_ expression as they maturate from DP to SP cells, making them more responsive to S1P, which is present at higher levels in blood allowing their egress from the thymus [7], [8], [10]. Since S1P_1_ is a G protein-coupled receptor (thus resembling to CXCR4) we hypothesized that SEMA3F could also block S1P_1_-induced migration of human thymocytes. We first analyzed S1P_1_ expression in human thymocytes and we observed higher levels of S1P_1_ expression on mature SP CD4^+^CD8^−^ and CD4^−^CD8^+^ compared with immature DP CD4^+^CD8^+^ thymocytes (Fig. 3a). Flow cytometry results correlated with real-time quantitative PCR of sorted subpopulations (data not shown). In a functional context, we observed that human thymocytes migrate towards S1P at 10 nM (Fig. 3b–c). In agreement with our hypothesis, SEMA3F also blocked S1P-induced migration (Fig. 3b–c). In this case, SEMA3F and S1P cell counts were below controls without stimuli in all experiments.

Pre-treatment of thymocytes with the blocking anti-NRP2 mAb partially reverted the inhibition of S1P chemoattraction caused by SEMA3F (Fig. 3d). In this case, cells migrate as controls (normalized to 1). This can be explained by the effect of SEMA3F on S1P-induced chemoattraction, which was strongest than the inhibition observed for CXCL12.

Furthermore, we analyzed the F-actin modulation of cytoskeleton on thymocytes following S1P stimulus. We observed a peak of actin polymerization after 15 s of stimulus (Fig. 3e), showing the ability of S1P_1_ to induce early steps in thymocyte migration as seen with CXCL12. When cells were pre-treated with SEMA3F, the S1P-induced actin polymerization was prevented and we even observed a tendency of actin depolymerization, in agreement with migration experiments.

### SEMA3F and NRP2 expression and function in T-cell acute lymphoblastic leukemia and lymphoma

**Figure 4:**
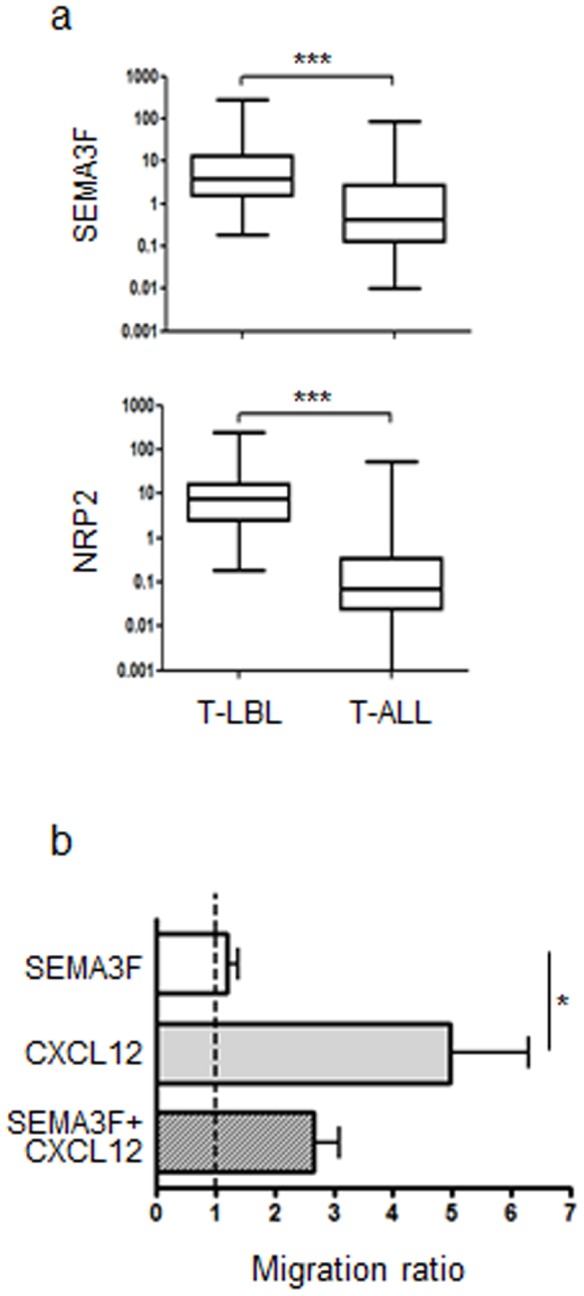
NRP2 and SEMA3F are expressed in T-ALL and T-LBL samples and modulate the migration of malignant cells. **a)** Box plots show the NRP2 and SEMA3F mRNA expression analyzed by real time quantitative PCR, compared with the control Abelson (Abl) gene in T-ALL (n =  136) and T-LBL (n =  37) samples. Results were analyzed by the non-parametric Wilcoxon Mann-Whitney test (***p<0.001). **b)** Bars represent migration of T-ALL cells in a transwell system. Results are represented by migration ratio, and controls without stimuli were normalized do 1.0. n = 4. Data are represented as means ± SEM. Results were analyzed by the unpaired Student's *t* test *p<0.05.

Based on our results regarding normal T cell precursors, we hypothesized that NRP2 and SEMA3F could also be expressed by malignant T cell precursors and have a role on lymphoblast migration which may explain various clinical patterns of disease presentation e.g. leukemic vs. lymphoma. We therefore analyzed mRNA expression of corresponding transcripts in T-cell acute lymphoblastic leukemia (T-ALL) and T-cell lymphoblastic lymphoma (T-LBL) samples. T-ALL and LBL are malignant proliferations of T cell precursors whose differentiation is arrested and are thought to originate in the thymus [21], [22]. We found that both T cell neoplasias expressed the transcripts, but NRP2 and SEMA3F were more highly expressed in T-LBL when compared with T-ALL samples (p<0.001) (Fig. 4a). In addition, the protein level in T-LBL biopsies, SEMA3F expression was stronger in intrathymic malignant cells when compared with the normal thymus or with T-LBL cells localized in blood and peripheral lymphoid organs such as lymph nodes as well as in non-lymphoid organs (data not shown).

It has been previously demonstrated that CXCR4 is expressed by malignant B and T-ALL, and the expression levels correlate with transmigration towards CXCL12 [23]. Therefore, we analyzed the effect of SEMA3F on spontaneous or CXCL12 induced migration. The corresponding migratory response of T-ALL cells was not substantial comparing with the one observed with normal thymocytes. Conversely, we observed a significant migration of malignant cells towards CXCL12. Importantly, the addition of SEMA3F partially prevented CXCL12-driven migration. We observed a 33% to 53% reduction of migration in the 4 samples analyzed (Fig. 4b).

We further asked whether T-ALL/LBL malignant cells also express S1P_1_. We observed that both neoplasias expressed S1P_1_ mRNA with no difference between T-ALL and T-LBL samples (Fig. S1a). Cell migration towards S1P was evaluated in only one case, in which we observed that cells did migrate towards S1P_1_ (1.8 ratio S1P_1_/control media) and that such migratory response was inhibited 78% when SEMA3F was added (Fig. S1b).

## Discussion

Herein, we show for the first time that NRP2 and SEMA3F are expressed in the human thymus and play a role in thymocyte migration. NRP2 expression varied depending on the maturation stage of the T cell precursors being downregulated in the more mature stages. Individually, SEMA3F had a repulsive effect on thymocytes, similar to data described for neurons [24], [25]. When added conjointly with the chemokine CXCL12 or the sphingolipid S1P, SEMA3F significantly prevented thymocyte migration induced by these molecules, and at one mechanism involved is the blockage in early steps of cytoskeleton reorganization, as ascertained by the effect upon actin polymerization.

Interestingly, the expression of NRP2 contrasted with the expression of S1P_1_ in human thymocytes. NRP2 is downregulated in SP cells whereas S1P_1_ is upregulated. Since S1P_1_ is essential for thymocyte egress [7] and SEMA3F can block S1P_1_-induced migration, one can argue that NRP2 downregulation could also be involved in thymocyte egress.

We have recently shown that other class 3 SEMA, the SEMA3A, also inhibited thymocyte migration towards CXCL12 and downregulated membrane expression of CXCR4 [26]. The inhibitory effect of SEMA3A was not observed towards the CCL21 chemokine, suggesting that the inhibition of thymocyte migration was specific for CXCL12. Herein, we observed that SEMA3F was able to inhibit thymocyte migration induced by the stimulus of other G-protein coupled receptor such as S1P_1_, which is essential for thymocyte egress [7]. It is important to note that the SEMA3A receptor NRP1 is expressed in 5.11±1.17% of thymocytes [14], whereas 89.84±3,57% of human thymocytes express NRP2. Strongest effects of SEMA3F could be in part explained by the differential expression patterns of NRPs.

We have proposed that thymocyte migration is a result of the action of several stimuli. Accordingly, each thymocyte responds migrating to a given direction, with a given velocity, which can change depending on the concentration and combination of each stimulus in each thymic region, as well as their capacity to respond via the corresponding specific receptors, in a process that we called multivectorial thymocyte migration [20]. In this context, SEMAs and NRPs can be placed as individual interactions (or vectors), and any alteration in their expression could alter thymocyte migration.

NRPs and SEMAs have been involved in different pathologies, mainly neurodevelopmental disorders and cancer. The expression levels of NRPs and SEMAs are altered in tumors of many types being related to tumor progression [27], [28], [29]. Interestingly, we also observed the expression of NRP2 and SEMA3F in neoplastic human T cell precursors, in the case of T-ALL and T-LBL. SEMA3F blocked the migration of T-ALL fresh samples towards CXCL12 and S1P, suggesting that malignant T cell precursors respond to SEMA3F similarly to normal thymocytes. Although this issue needs further analysis, our results on normal thymocytes strongly support this hypothesis.

T-ALL and T-LBL are considered as different forms of a single disease [30]. However, although frequently accompanied by a mediastinal mass and bulky adenopathy, T-ALL is characterized by greater than 25% bone marrow involvement. In contrast, T-LBL is most typically characterized by a large anterior mediastinal mass, with discrete bone marrow involvement (<25%). Since T-LBL expressed higher levels of both molecules when compared with T-ALL samples, it is conceivable to hypothesize that the SEMA3F/NRP2 axis could play a role in malignant T cell precursors homing.

In conclusion, in this paper we have shown that the SEMA3F/NRP2 axis is involved in thymocytes migration and as such should play a critical role in central immune regulation and in the physiopathology of neoplastic disorders of thymocytes. It is tempting to speculate that our findings may provide rationale to investigate the role of drugs that could interact with this pathway for example in the treatment of some immune as well as neoplastic disorders involving normal and malignant thymocytes.

## Supporting Information

Figure S1 **SEMA3F modulates S1P-induced migration of T-ALL and T-LBL malignant cells.** **a)** Box plot shows S1P1 mRNA expression analyzed by real time quantitative PCR, compared with the control Abelson (Abl) gene in T-ALL (n =  136) and T-LBL (n =  37) samples. Results were analyzed by the non-parametric Wilcoxon Mann-Whitney test. **b)** Bars represent migration of a T-ALL sample in a transwell system. Results are represented by migration ratio, and control without stimuli was normalized do 1.0.
(TIF)

## References

[1] Savino W, Dalmau SR, Dealmeida VC (2000) Role of extracellular matrix-mediated interactions in thymocyte migration. Dev Immunol 7: 279–291. 11097218 10.1155/2000/60247 PMC2276042

[2] Poznansky MC, Olszak IT, Evans RH, Wang Z, Foxall RB, et al (2002) Thymocyte emigration is mediated by active movement away from stroma-derived factors. J Clin Invest 109: 1101–1110. 11956248 10.1172/JCI13853 PMC150941

[3] Vianello F, Kraft P, Mok YT, Hart WK, White N, et al (2005) A CXCR4-dependent chemorepellent signal contributes to the emigration of mature single-positive CD4 cells from the fetal thymus. J Immunol 175: 5115–5125. 16210615 10.4049/jimmunol.175.8.5115

[4] Annunziato F, Romagnani P, Cosmi L, Lazzeri E, Romagnani S (2001) Chemokines and lymphopoiesis in human thymus. Trends Immunol 22: 277–281. 11323287 10.1016/s1471-4906(01)01889-0

[5] Dzhagalov I, Phee H (2012) How to find your way through the thymus: a practical guide for aspiring T cells. Cell Mol Life Sci 69: 663–682. 21842411 10.1007/s00018-011-0791-6 PMC3271173

[6] Halkias J, Melichar HJ, Taylor KT, Ross JO, Yen B, et al (2013) Opposing chemokine gradients control human thymocyte migration in situ. J Clin Invest 123: 2131–2142. 23585474 10.1172/JCI67175 PMC3635739

[7] Matloubian M, Lo CG, Cinamon G, Lesneski MJ, Xu Y, et al (2004) Lymphocyte egress from thymus and peripheral lymphoid organs is dependent on S1P receptor 1. Nature 427: 355–360. 14737169 10.1038/nature02284

[8] Allende ML, Dreier JL, Mandala S, Proia RL (2004) Expression of the sphingosine 1-phosphate receptor, S1P1, on T-cells controls thymic emigration. J Biol Chem 279: 15396–15401. 14732704 10.1074/jbc.M314291200

[9] Schwab SR, Pereira JP, Matloubian M, Xu Y, Huang Y, et al (2005) Lymphocyte sequestration through S1P lyase inhibition and disruption of S1P gradients. Science 309: 1735–1739. 16151014 10.1126/science.1113640

[10] Chiba K, Matsuyuki H, Maeda Y, Sugahara K (2006) Role of sphingosine 1-phosphate receptor type 1 in lymphocyte egress from secondary lymphoid tissues and thymus. Cell Mol Immunol 3: 11–19. 16549044

[11] Yopp AC, Ledgerwood LG, Ochando JC, Bromberg JS (2006) Sphingosine 1-phosphate receptor modulators: a new class of immunosuppressants. Clin Transplant 20: 788–795. 17100731 10.1111/j.1399-0012.2006.00570.x

[12] de Wit J, Verhaagen J (2003) Role of semaphorins in the adult nervous system. Prog Neurobiol 71: 249–267. 14687984 10.1016/j.pneurobio.2003.06.001

[13] Kruger RP, Aurandt J, Guan KL (2005) Semaphorins command cells to move. Nat Rev Mol Cell Biol 6: 789–800. 16314868 10.1038/nrm1740

[14] Lepelletier Y, Smaniotto S, Hadj-Slimane R, Villa-Verde DM, Nogueira AC, et al (2007) Control of human thymocyte migration by Neuropilin-1/Semaphorin-3A-mediated interactions. Proc Natl Acad Sci U S A 104: 5545–5550. 17369353 10.1073/pnas.0700705104 PMC1838472

[15] Mendes-da-Cruz DA, Lepelletier Y, Brignier AC, Smaniotto S, Renand A, et al (2009) Neuropilins, semaphorins, and their role in thymocyte development. Ann N Y Acad Sci 1153: 20–28. 19236324 10.1111/j.1749-6632.2008.03980.x

[16] Stepanova OI, Krylov AV, Lioudyno VI, Kisseleva EP (2007) Gene expression for VEGF-A, VEGF-C, and their receptors in murine lymphocytes and macrophages. Biochemistry (Mosc) 72: 1194–1198. 18205601 10.1134/s0006297907110041

[17] Takahashi K, Ishida M, Hirokawa K, Takahashi H (2008) Expression of the semaphorins Sema 3D and Sema 3F in the developing parathyroid and thymus. Dev Dyn 237: 1699–1708. 18489001 10.1002/dvdy.21556

[18] Giger RJ, Urquhart ER, Gillespie SK, Levengood DV, Ginty DD, et al (1998) Neuropilin-2 is a receptor for semaphorin IV: insight into the structural basis of receptor function and specificity. Neuron 21: 1079–1092. 9856463 10.1016/s0896-6273(00)80625-x

[19] Chalasani SH, Sabelko KA, Sunshine MJ, Littman DR, Raper JA (2003) A chemokine, SDF-1, reduces the effectiveness of multiple axonal repellents and is required for normal axon pathfinding. J Neurosci 23: 1360–1371. 12598624 10.1523/JNEUROSCI.23-04-01360.2003 PMC6742262

[20] Mendes-da-Cruz DA, Smaniotto S, Keller AC, Dardenne M, Savino W (2008) Multivectorial abnormal cell migration in the NOD mouse thymus. J Immunol 180: 4639–4647. 18354187 10.4049/jimmunol.180.7.4639

[21] Crist WM, Shuster JJ, Falletta J, Pullen DJ, Berard CW, et al (1988) Clinical features and outcome in childhood T-cell leukemia-lymphoma according to stage of thymocyte differentiation: a Pediatric Oncology Group Study. Blood 72: 1891–1897. 3058229

[22] Uckun FM, Sensel MG, Sun L, Steinherz PG, Trigg ME, et al (1998) Biology and treatment of childhood T-lineage acute lymphoblastic leukemia. Blood 91: 735–746. 9446631

[23] Crazzolara R, Kreczy A, Mann G, Heitger A, Eibl G, et al (2001) High expression of the chemokine receptor CXCR4 predicts extramedullary organ infiltration in childhood acute lymphoblastic leukaemia. Br J Haematol 115: 545–553. 11736934 10.1046/j.1365-2141.2001.03164.x

[24] Atwal JK, Singh KK, Tessier-Lavigne M, Miller FD, Kaplan DR (2003) Semaphorin 3F antagonizes neurotrophin-induced phosphatidylinositol 3-kinase and mitogen-activated protein kinase kinase signaling: a mechanism for growth cone collapse. J Neurosci 23: 7602–7609. 12930799 10.1523/JNEUROSCI.23-20-07602.2003 PMC6740747

[25] Pascual M, Pozas E, Soriano E (2005) Role of class 3 semaphorins in the development and maturation of the septohippocampal pathway. Hippocampus 15: 184–202. 15386596 10.1002/hipo.20040

[26] Garcia F, Lepelletier Y, Smaniotto S, Hadj-Slimane R, Dardenne M, et al (2011) Inhibitory effect of semaphorin-3A, a known axon guidance molecule, in the human thymocyte migration induced by CXCL12. J Leukoc Biol 10.1189/jlb.0111031 21878545

[27] Ellis LM (2006) The role of neuropilins in cancer. Mol Cancer Ther 5: 1099–1107. 16731741 10.1158/1535-7163.MCT-05-0538

[28] Neufeld G, Kessler O (2008) The semaphorins: versatile regulators of tumour progression and tumour angiogenesis. Nat Rev Cancer 8: 632–645. 18580951 10.1038/nrc2404

[29] Gaur P, Bielenberg DR, Samuel S, Bose D, Zhou Y, et al (2009) Role of class 3 semaphorins and their receptors in tumor growth and angiogenesis. Clin Cancer Res 15: 6763–6770. 19887479 10.1158/1078-0432.CCR-09-1810

[30] Harris NL, Jaffe ES, Diebold J, Flandrin G, Muller-Hermelink HK, et al (1999) The World Health Organization classification of neoplastic diseases of the hematopoietic and lymphoid tissues. Report of the Clinical Advisory Committee meeting, Airlie House, Virginia, November, 1997. Ann Oncol 10: 1419–1432. 10643532 10.1023/a:1008375931236

