# Supplementary figures and images for: Semaphorin 3F and Neuropilin-2 Control the Migration of Human T-Cell Precursors
Source: PLoS One. 2014 Jul 28;9(7):e103405. doi: 10.1371/journal.pone.0103405 (PMC4113369; doi:10.1371/journal.pone.0103405)

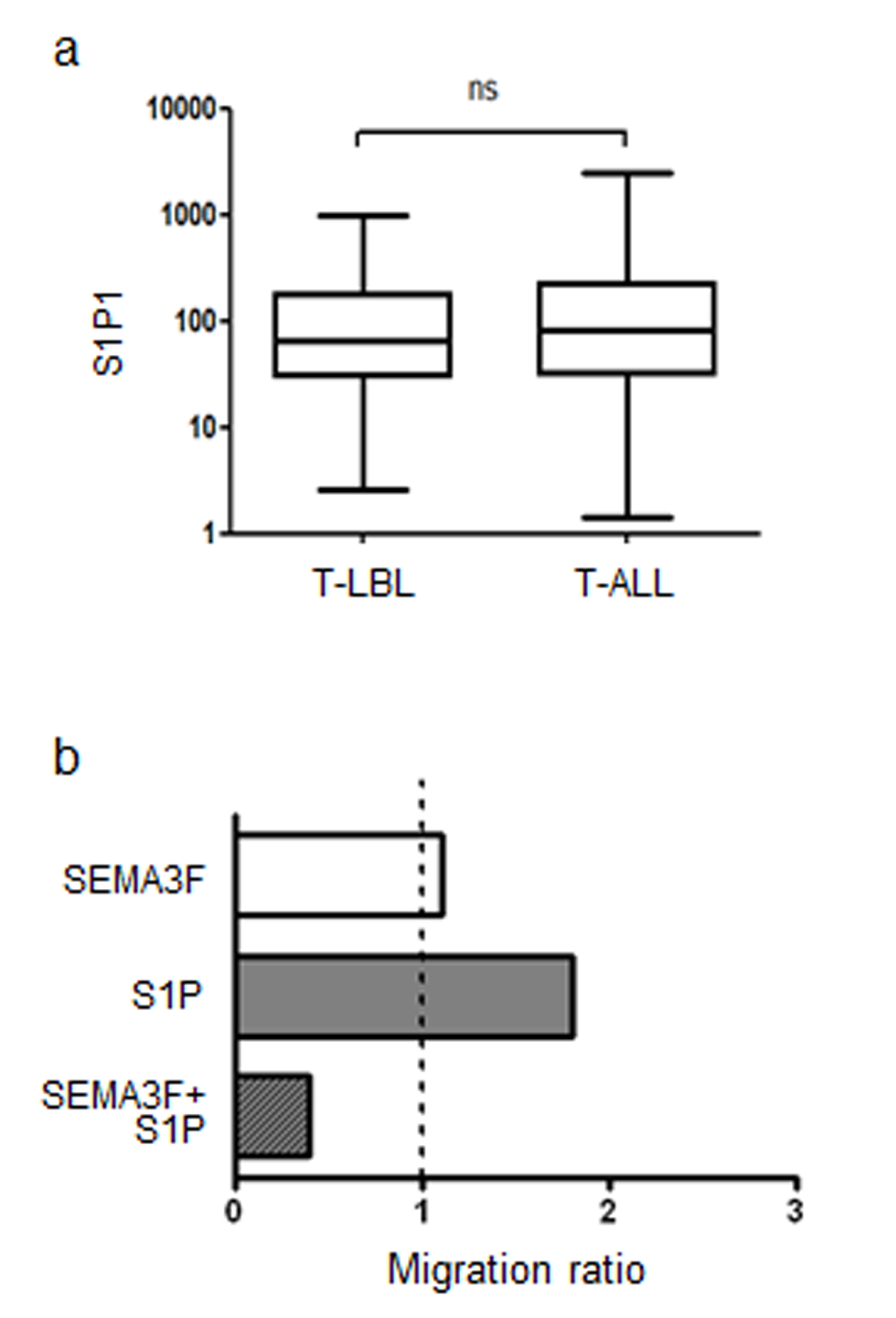

Supplement: Figure S1 — SEMA3F modulates S1P-induced migration of T-ALL and T-LBL malignant cells. a) Box plot shows S1P1 mRNA expression analyzed by real time quantitative PCR, compared with the control Abelson (Abl) gene in T-ALL (n = 136) and T-LBL (n = 37) samples. Results were analyzed by the non-parametric Wilcoxon Mann-Whitney test. b) Bars represent migration of a T-ALL sample in a transwell system. Results are represented by migration ratio, and control without stimuli was normalized do 1.0. (TIF) [file pone.0103405.s001.tif]
